# Krankheitsvorstellungen von Patient*innen mit berufsbedingten Handekzemen vor dem Hintergrund soziodemografischer Merkmale: Eine qualitative Studie im Rahmen einer tertiären Individualpräventionsmaßnahme

**DOI:** 10.1007/s11553-023-01039-2

**Published:** 2023-04-29

**Authors:** Andreas Hansen, Annika Wilke, Swen Malte John, Anna-Sophie Buse

**Affiliations:** 1 Abteilung Dermatologie, Umweltmedizin und Gesundheitstheorie, Institut für Gesundheitsforschung und Bildung, Universität Osnabrück, Am Finkenhügel 7a, 49076 Osnabrück, Deutschland; 2 Institut für interdisziplinäre Dermatologische Prävention und Rehabilitation (iDerm), an der Universität Osnabrück, Am Finkenhügel 7a, 49076 Osnabrück, Deutschland

**Keywords:** Subjektive Krankheitstheorien, Berufsbedingte Hauterkrankungen, Kontaktekzem, Qualitative Inhaltsanalyse, Leitfadeninterviews, Lay theories, Occupational skin disease, Contact Dermatitis, Qualitative content analysis, Guided interviews

## Abstract

**Hintergrund:**

Berufsbedingte Hauterkrankungen treten in vielen Berufen auf. Häufig handelt es sich um irritative und/oder allergische Handekzeme. Zu den häufigsten betroffenen Berufsgruppen zählen z. B. Pflegekräfte und Metallarbeiter*innen. Hinsichtlich soziodemografischer Merkmale (Alter, Berufe, Geschlecht) ist das Patientenklientel insgesamt sehr heterogen. Individualpräventive ambulante und stationäre Maßnahmen zielen u. a. darauf ab, das Selbstmanagement mit der Erkrankung zu verbessern (z. B. Veränderung des Hautschutzverhaltens) und ein erkrankungsbedingtes Ausscheiden aus dem Beruf zu verhindern. Subjektive Krankheitstheorien spielen bei der Erreichung dieser Ziele eine wichtige Rolle, so dass es das Ziel der Studie war, diese subjektiven Theorien unter besonderer Beachtung soziodemografischer Merkmale zu erfassen und zu analysieren.

**Methode:**

Es wurden 36 leitfadengestützte qualitative Interviews mit Patient*innen einer stationären Individualpräventionsmaßnahme geführt. Die Auswertung erfolgte mittels inhaltlich strukturierender qualitativer Inhaltsanalyse. Hauptkategorien wurden deduktiv anhand des theoretischen Rahmens (Common-Sense-Modell) gebildet, Subkategorien anhand des Materials (induktiv).

**Ergebnisse:**

Interviewtranskripte von 35 Patient*innen wurden analysiert (Geschlecht: weiblich = 18, männlich = 17/Alter: minimal: 22 Jahre, maximal: 63 Jahre). Dabei ließen sich alle Dimensionen des Rahmenmodells mit verschiedenen Subkategorien abbilden. Die vermuteten bzw. wahrgenommenen Ursachen waren überwiegend komplex. Hinsichtlich der Altersgruppen und Berufe konnten teilweise Unterschiede festgestellt werden. So waren z. B. Aussagen, die auf eine höhere Selbstwirksamkeit in Bezug auf die Kontrollierbarkeit des Handekzems hindeuteten eher bei interviewten Pflegekräften zu finden, als bei den Befragten der Metallberufe.

**Schlussfolgerungen:**

Die Wahrnehmung komplexer Ursachen spiegelt die oftmals multifaktorielle Krankheitsentstehung wider (z. B. irritative Handekzeme bei atopischer Disposition). Die z. T. vorgefundenen Unterschiede zwischen Berufs- und Altersgruppen verdeutlichen, dass involvierte Berufsgruppen (z. B. Ärzt*innen, Therapeut*innen) bei der Berücksichtigung der subjektiven Krankheitsvorstellungen auch ein Zusammenspiel mit soziodemografischen Aspekten erwägen sollten.

## Abstract (en)

**Background:**

Occupational, inflammatory skin diseases are a common work-related disease. Often, these diseases are irritative and/or allergic hand eczema. Affected groups include, for example, nursing staff and metal workers. Individual outpatient and inpatient prevention programs aim at improving self-management of the disease (i.e., improving skin protection behavior) and preventing affected workers from job loss due to their illness. The common-sense model is a widely used framework to explain the patients’ illness perceptions of their disease. Qualitative research methods are appropriate means to explore illness perceptions. As the group of patients suffering from occupational skin diseases is heterogeneous (age, professions, gender), it seems necessary to focus on sociodemographic characteristics.

**Methods:**

After conducting guided interviews with 36 patients of a tertiary individual prevention program, the analysis of 35 interview transcripts was carried out using qualitative content analysis. After building main categories deductively, based on the theoretical framework (Common-Sense Model), subcategories were formed inductively, based on the data material.

**Results:**

Transcripts of 35 patients were analyzed (18 women, 17 men, age: 22–63 years). All dimensions of the theoretical framework could be mapped in different subcategories. Here, especially the complexity of suspected and/or perceived causes named by the participants became apparent. With regard to age groups and occupations, some differences were found in the material. For example, statements indicating self-efficacy with regard to the controllability of hand eczema were more likely to be found among nursing staff than among metal workers.

**Conclusion:**

The complexity of the perceived causes reflects the multifactorial etiology of the disease (e.g., irritative hand eczema with atopic disposition). In some cases, differences between occupational and age groups became apparent. This makes clear that occupational groups involved in individual prevention (e.g., physicians, therapists) should also reflect the potential impact of sociodemographic aspects when considering subjective perceptions of disease.

## Hintergrund und Fragestellung

Zusammenhänge zwischen Arbeit und der Gesundheit von Arbeitnehmer*innen sind vielfach untersucht und belegt [17]. In den vergangenen Jahrzehnten sind physische Gefahren zugunsten psychosozialer Belastungen vermehrt in den Hintergrund getreten [25]. In Abhängigkeit von der beruflichen Tätigkeit bestehen jedoch weiterhin an vielen Arbeitsplätzen physische Gefährdungen (bspw. infolge von Lärm, muskuloskelettalen Belastungen und Hautbelastungen). Diese können zu beruflich (mit)verursachten Erkrankungen und zu Berufskrankheiten gemäß Berufskrankheitenverordnung (BKV) führen [3, 8]. Berufsbedingte, entzündliche Hauterkrankungen im Sinne der Berufskrankheiten-Nr. 5101 (BK-Nr. 5101) treten in verschiedensten Berufsgruppen auf und gehören seit Jahrzehnten zu den häufigsten Erkrankungen, die bei den Trägern der Gesetzlichen Unfallversicherung angezeigt werden [8, 10]. So wurden im Jahr 2021 mehr als 17.000 Verdachtsfälle der BK-Nr. 5101 gemeldet [8]. Eine weitaus höhere Dunkelziffer der nicht gemeldeten Erkrankungen wird angenommen [10]. Der überwiegende Anteil dieser Hauterkrankungen entfällt auf irritativ und/oder allergisch versursachte Handekzeme [13, 35]. Berufsbedingte Handekzeme treten insbesondere in Berufen mit einem hohen Anteil an Feuchtarbeit häufig auf. So zählen u. a. Friseur*innen, Gesundheits- und Krankenpfleger*innen sowie Berufstätige in Bau- und Metallberufen zu den häufig betroffenen Berufsgruppen [2, 10, 21, 23, 24]. Sie führen oft zu einer eingeschränkten Lebensqualität der Betroffenen [4], zu hohen Kosten [11] und können Arbeitsunfähigkeit und eine Berufsaufgabe nach sich ziehen [5]. Ambulante und stationäre Maßnahmen im Sinne der sekundären bzw. tertiären Individualprävention zählen seit rund 2 Jahrzehnten zur Regelversorgung der Unfallversicherungsträger für berufsbedingt hauterkrankte Personen [5, 33]. Zu den Zielen dieser Maßnahmen gehören u. a. die Optimierung der Therapie, die Verbesserung der Hautschutzmaßnahmen, der Erwerb von krankheitsspezifischem Wissen sowie insbesondere die Vermeidung einer erkrankungsbedingten Berufsaufgabe [33]. Die Gruppe der Teilnehmenden an ambulanten und stationären Präventionsmaßnahmen ist sehr heterogen: Frauen und Männer aller berufstätigen Altersgruppen aus verschiedensten Berufsgruppen nehmen diese Präventionsangebote in Anspruch [5, 34]. Pflegekräfte und auch in Metallberufen tätige Arbeitnehmer*innen zählen zu den am häufigsten vertretenen Berufsgruppen in der stationären Individualprävention [6, 35]. Ein erkrankungsbedingtes Ausscheiden aus dem Beruf kann den Fachkräftemangel weiter verschärfen, dessen Folgen insbesondere in medizinisch-pflegerischen Berufen nicht erst seit Beginn der SARS-CoV-2-Pandemie („severe acute respiratory syndrome coronavirus 2“) diskutiert werden. So zählten im Juni 2022 gerade die Berufsgruppen „Medizinische Gesundheitsberufe“ und „Metallerzeugung, -bearbeitung, Metallbau“ zu den sechs Berufsgruppen, in denen mehr als 50.000 offene Arbeitsstellen bei der Bundesagentur für Arbeit gemeldet waren [7]. Vor diesem Hintergrund wird die gesamtgesellschaftliche Relevanz beruflicher Handekzeme und der darauf bezogenen Präventionsmaßnahmen besonders deutlich.

Ein adäquates Hautschutzverhalten und damit die Verhaltensprävention ist ein wichtiger Baustein in der Prävention dieser Erkrankungen und ein wichtiger Einflussfaktor auf das Krankheitsverhalten bzw. das präventive Verhalten der Patient*innen ist die Sichtweise auf ihre eigene Erkrankung. Nimmt eine Person körperliche Beschwerden wahr, werden diese auf der Grundlage bisheriger Erfahrungen, des eigenen Vorwissens und vielfach auch im Austausch mit Bezugspersonen (z. B. Partner, Freunde) hinsichtlich ihres Erkrankungswertes und einer möglichen Bedrohung eingeordnet [17]. Diese subjektive Einschätzung geschieht bereits vor und unabhängig von einer Einschätzung und Diagnostik durch (medizinische) Expert*innen [14, 17]. Ferner führt nicht jede in diesem Rahmen getroffene Laiendiagnose notwendigerweise zum Aufsuchen ärztlicher Expert*innen [17]. Dies zeigen bspw. alltägliche Beschwerden, wie Kopfschmerzen und Erkältungen, die oftmals ausschließlich im sogenannten „Laiengesundheitssystem“ diagnostiziert und behandelt werden [17]. Menschen entwickeln jedoch auch dann, wenn sie erkranken und eine ärztliche Diagnose ihrer Erkrankung erhalten, subjektive Vorstellungen über ihre Erkrankung (z. B. hinsichtlich der Entstehungsursachen und des möglichen Verlaufs; [17, 19, 20]). Dabei fließen insbesondere die eigenen Erfahrungen in die Krankheitsvorstellungen mit ein [19]. Auch wenn intraindividuell Parallelen zu der Entwicklung wissenschaftlicher Erklärungen über Erkrankungen konstatiert werden können [19], sind subjektive Theorien oftmals nicht kongruent zu wissenschaftlichen Theorien [14, 17]. Da die subjektiven Krankheitstheorien für die Auseinandersetzung mit der eigenen Erkrankung essenziell [19] und handlungsleitend sind [17], haben sie jedoch einen wesentlichen Einfluss auf das Krankheitsverhalten der Betroffenen, was sich bspw. in der Wahrnehmung von Symptomen äußert, aber auch in der Adhärenz [16]. Untersuchungen zeigen, dass soziodemografische Aspekte bei der Ausprägung subjektiver Krankheitstheorien bedeutsam sein können. So sind z. T. kulturelle Unterschiede hinsichtlich der Wahrnehmung von Krankheitsursachen [15, 32] und bspw. bei Krebserkrankungen geschlechtsspezifisch unterschiedliche Ausprägungen feststellbar [15].

Das Common-Sense-Modell (CSM) von Leventhal et al. [26] ist ein weit verbreitetes und häufig rezipiertes Theorierahmenmodell, um zu erklären, wie Personen auf gesundheitliche Bedrohungen reagieren [9]. Der zugrunde liegende Gedanke des Modells ist, dass die Wahrnehmung einer gesundheitlichen Bedrohung zu einem Prozess der Selbstregulierung führt, um die Gesundheitsbedrohung zu bewältigen [26]. Die einzelnen Dimensionen (kognitive Repräsentationen) des CSM sind: Identität, Verlauf, Konsequenzen, Kontrollierbarkeit und Ursache [9, 26]. Der auf Grundlage des CSM entwickelte Illness Perception Questionnaire (IPQ; [36]) sowie die Revisionsversion (IPQ‑R; [28]) messen die verschiedenen kognitiven Dimensionen sowie mit seiner Erweiterung auch emotionale Komponenten des Selbstregulationsmodells [28]. Subjektive Krankheitstheorien von beruflich Hauterkrankten wurden jedoch bisher nur in wenigen qualitativen Studien untersucht [1, 27, 30, 37]. Um tiefergehende Aussagen betroffener Patient*innen zu erhalten und die komplexen Theorien zu erfassen, eignen sich insbesondere qualitative Erhebungsmethoden [17, 29]. Bathe et al. [1] haben im Rahmen ihrer qualitativen Studie bereits auf das CSM rekurriert, nutzten jedoch als Rahmen eine Synthese verschiedener Theoriemodelle. Zudem wurden die im IPQ‑R enthaltenen Dimensionen „Emotionale Repräsentationen“ und „Kohärenz“ nicht aufgegriffen [1]. Auf soziodemografische Aspekte (bspw. das Alter) wurde in vorherigen qualitativen Untersuchungen verwiesen [1], sie standen jedoch nicht im Mittelpunkt. Vor diesem Hintergrund kann die Exploration subjektiver Krankheitstheorien einen wesentlichen Beitrag für individualpräventive Maßnahmen in der Berufsdermatologie leisten. Mit Bezug auf die sehr heterogene Patient*innengruppe (z. B. verschiedene Alters- und Berufsgruppen) liegt der Fokus dieses Artikels auf möglichen Besonderheiten hinsichtlich soziodemografischer Merkmale.

### Fragestellung

Inwiefern sind bei Patient*innen mit berufsbedingten Handekzemen vor dem Hintergrund des CSM Besonderheiten bei der Ausprägung subjektiver Krankheitstheorien in Bezug auf Alters- und Berufsgruppen feststellbar?

## Methodik

### Studiendesign

Im Rahmen der vorliegenden Studie wurden leitfadengestützte Interviews mit Patient*innen einer Maßnahme der stationären Individualprävention durchgeführt. Den theoretischen Rahmen bildeten das CSM-Modell [26] und der darauf aufbauende IPQ‑R [28]. Die Prüfung der Studie erfolgte durch die Ethikkommission der Universität Osnabrück und wurde positiv beschieden (Votum 13/2020).

### Leitfadenentwicklung

Auf Grundlage des CSM [26] bzw. des IPQ‑R [28] wurde ein teilstrukturierter Leitfaden entwickelt (Tab. 1). Zudem wurde die erzählgenerierende Einstiegsfrage „Erzählen Sie doch mal, wie das so war mir Ihrer Haut, in Ihrem bisherigen Leben“ formuliert. Zur Überprüfung der Verständlichkeit des Leitfadens führten die Interviewer*innen (AH und AB) vorab jeweils drei Pretestinterviews durch. Einzelne Fragen des Interviewleitfadens sowie die fakultativen Vertiefungsfragen wurden auf dieser Grundlage im Hinblick auf eine bessere Verständlichkeit angepasst.

**Tab. 1:** Interviewleitfaden

*Erzählimpuls:* Erzählen Sie doch mal, wie das so war mir Ihrer Haut, in Ihrem bisherigen Leben?	*Immanente Nachfragen *(zu Themen, die bereits angesprochen aber noch nicht ausgeführt wurden): Sie haben eben erwähnt, dass … … können Sie das nochmal näher erklären/beschreiben? … wie ist/war das für Sie?
*Exmanente Nachfragen* (zu Themen, die noch nicht angesprochen wurden, ggf. mit dem Paraphrasieren von bereits gesagtem kombinieren):	
*Identität* (z. B. Erkennung und Wahrnehmung von Symptomen):	Woran haben Sie gemerkt, dass Ihre Haut anders ist/sich verändert hat? Wie sieht Ihre Haut an den Händen aus? Beschreiben Sie doch mal die Veränderungen an Ihrer Haut. Inwiefern haben Sie weitere Veränderungen an Ihrem Körper wahrgenommen?
*Verlauf *(z. B. Erwartungen über die Dauer und den Verlauf der Erkrankung, z. B. chronisch, zyklisch, akut) ggf. im Interview mit Frage zur Ursache kombinierbar:	Wenn Sie an die letzten Jahre zurückdenken, wie entwickelte sich Ihre Haut? Optionale Nachfrage (wenn vorher nicht von Interviewer angesprochen): Wie lange dauerten die Phasen an? Wann war das? Wie stellen Sie sich vor, wird sich Ihre Erkrankung in der Zukunft entwickeln?
*Kontrollierbarkeit* (z. B. Annahmen zur Beeinflussbarkeit der Erkrankung durch eigenes Verhalten oder therapeutische Interventionen):	Was machen Sie, wenn sich Ihre Haut verändert/entzündet ist … (Individuell - je nach Gesprächsverlauf - anpassen)? Optionale Nachfrage: Wenn Sie an Ihre ärztlichen Behandlungen zurückdenken, was wurde gemacht, wenn Ihre Haut verändert/entzündet war … (individuell - je nach Gesprächsverlauf - anpassen)?
*Konsequenzen *(z. B. Annahmen zu den kurz- und langfristigen Folgen der Erkrankung, z. B. psychisch, sozial, emotional, physisch und/oder ökonomisch):	Wenn Sie an Ihren Alltag denken, welche Rolle spielen da die Hautveränderungen?
*Emotionale Repräsentation:*	Inwiefern lösen Ihre Hautveränderungen Gefühle bei Ihnen aus?
*Kohärenz; Verstehbarkeit/Erklärbarkeit:*	Inwiefern sind Ihre Hautveränderungen für Sie nachvollziehbar?

### Sampling und Rekrutierung

Im Sinne eines „purposive sampling“ wurde ein qualitativer Stichprobenplan entwickelt [31], der als ein zentrales Kriterium beinhaltete, Frauen und Männer in etwa gleichverteilt aus allen Altersgruppen im erwerbsfähigen Alter zu akquirieren. Die Altersgruppen wurden hierbei auf drei Gruppen aufgeteilt (18–35, 36–50, 51–65 Jahre). Ferner wurde aufgrund der Prävalenz avisiert, dass insbesondere Gesundheitsberufe und körpernahe Dienstleistungsberufe (z. B. Pflegeberufe, Friseurhandwerk) sowie Metallberufe und weitere handwerkliche Berufe (z. B. CNC-Maschinenbedienung, Kfz-Berufe) vertreten sein sollten. Als weitere Gruppe wurden kontrastierend Teilnehmende aus Berufen in den Stichprobenplan aufgenommen, in denen berufliche Hautbelastungen im Sinne der BK-Nr. 5101 als gering eingeschätzt werden (z. B. Tätige im Einzelhandel). Die Auswahl möglicher Interviewpartner*innen erfolgte durch zwei Wissenschaftler*innen (AH und AB). Die potenziellen Teilnehmenden wurden während einer 3‑wöchigen stationären Maßnahme der Individualprävention von den Interviewer*innen im Anschluss an Patientenschulungen (Hautschutzseminare) angesprochen. In diesem Rahmen wurde der Hintergrund der Studie kurz erläutert und ein grundsätzliches Interesse zur Teilnahme erfragt. Die Freiwilligkeit wurde explizit thematisiert. Sofern eine grundsätzliche Teilnahmebereitschaft vorlag, erhielten die Teilnehmenden eine schriftliche Probandenaufklärung und eine Einwilligungserklärung. Die Pseudonymisierung der Interviewdaten wurde in beiden Dokumenten beschrieben. Eine unterschriebene Einwilligungserklärung lag jeweils vor der Interviewdurchführung vor. Der Interviewtermin wurde von der Interviewerin bzw. dem Interviewer mit den jeweiligen Teilnehmenden individuell vereinbart.

### Interviewdurchführung

Die Interviews wurden im Zeitraum von Mai bis Oktober 2021 geführt (AB: 19 Interviews, AH: 17 Interviews). Die Befragung erfolgte während des stationären Aufenthalts. Alle Interviews wurden innerhalb der Klinik unter Einhaltung der damals geltenden Hygienevorgaben (Maske und Abstand) durchgeführt. Zur Vermeidung von Störungen wurde ein entsprechender Informationszettel an der Außenseite der Tür angebracht. Die Aufnahme der Interviews erfolgte mit einem digitalen Diktiergerät (Olympus DM-770, Olympus, Hamburg, Deutschland), das auf einem Tisch zwischen beide Interviewpartner*innen gelegt wurde.

### Auswertung der Interviews

Die Interviews wurden zunächst von einem professionellen Transkriptionsdienstleister (abtipper.de, Digitalmeister GmbH, Hannover, Deutschland) nach einem vereinfachten Transkriptionssystem vollständig und wörtlich transkribiert und [12]. Die Transkripte wurden im Anschluss vollständig überprüft (AB und AH) und pseudonymisiert. Die Auswertung erfolgte mittels inhaltlich strukturierender qualitativer Inhaltsanalyse (QIA) unter Zuhilfenahme des Programms MAXQDA 2018 (Verbi, Berlin, Deutschland; [22]). Hauptkategorien wurden a priori (deduktiv) anhand des IPQ‑R entwickelt [26]. Vor der Kodierung wurden Kategoriendefinitionen und Kodierregeln von beiden Wissenschaftler*innen (AH und AB) konsentiert. Im Sinne einer Intercoder-Übereinstimmung wurden zunächst vier Interviewtranskripte unabhängig von beiden Forscher*innen anhand der deduktiven Kategorien kodiert und anschließend verglichen. Bei divergierenden Einschätzungen erfolgte eine Konsensbildung und bei Bedarf eine Anpassung der Kodierregeln. Ferner wurde das Kategoriensystem im Rahmen einer interdisziplinären Forschungswerkstatt (methodischer Schwerpunkt: QIA) diskutiert. Darauf aufbauend kodierten beide Wissenschaftler*innen je zur Hälfte die weiteren Interviews. Anschließend wurden Subkategorien in einem induktiven Prozess durch beide Autor*innen gemeinsam am Datenmaterial entwickelt. Nach rund einem Drittel der Interviews war eine empirische Sättigung erreicht, so dass keine weiteren Subkategorien hinzukamen. Anschließend erfolgte erneut anhand von vier gleichen Interviews eine unabhängige Kodierung mit darauf folgender Konsensbildung. Die weiteren Interviews wurden erneut je zur Hälfte von beiden Wissenschaftler*innen anhand der induktiv gebildeten Kategorien kodiert. Im Fall von unklaren Kodierungen erfolgte eine erneute Konsentierung.

## Ergebnisse

### Sample

Es wurden insgesamt 36 Interviews mit stationären Patient*innen geführt. Da sich bei einem Interviewten im Verlauf der Diagnostik zeigte, dass kein Zusammenhang zwischen der beruflichen Tätigkeit und der Hauterkrankung bestand, wurde das betreffende Interview nicht in die Auswertung aufgenommen. Die Gesamtdauer der Interviews betrug rund 9,5 h mit einer durchschnittlichen Interviewdauer von 16,14 min (Tab. 2). Mit einer Anzahl von 18 weiblichen und 17 männlichen Interviewten bestand eine Gleichverteilung in Bezug auf das Geschlecht.

**Tab. 2:** Interviewdaten im Überblick

*n*	35
Durchschnittsalter	43 Jahre (min: 22 Jahre; max.: 63 Jahre)
Weiblich	*n* = 18
Männlich	*n* = 17
Gesamtdauer der Interviews	09:28:13 h
Durchschnittliche Interviewdauer	16,14 min (min: 6,12 min; max: 41,34 min)
Gesundheitsberufe und körpernahe Dienstleistungsberufe	*n* = 13
Metallberufe und weitere handwerkliche Berufe	*n* = 19
Verkäufer*innen	*n* = 3
Alter 18–35 Jahre	*n* = 14
Alter 36–50 Jahre	*n* = 7
Alter 51–65 Jahre	*n* = 14

### Kategorien

Im Rahmen des Auswertungsprozesses wurden zu allen a priori gebildeten Hauptkategorien (*Ursache, Verlauf, Identität, Kontrollierbarkeit, Konsequenzen, Kohärenz, Emotionale Repräsentationen*) induktiv Subkategorien sowie teilweise Subsubkategorien gebildet.

Da die Wahrnehmung von Einflussfaktoren auf die Entstehung und den Verlauf des Handekzems eine wesentliche Grundlage dafür bildet, welche Präventions- und Therapiemaßnahmen aus Sicht der Hauterkrankten als adäquat erachtet werden [9, 18], stellt sich die Hauptkategorie Ursache als eine basale Dimension dar. Vor dem Hintergrund der besonderen Relevanz werden daher insbesondere zentrale Ergebnisse der Analyse dieser Kategorie berichtet. Es werden zudem Auffälligkeiten dahingehend dargestellt, wie die Befragten ihre Vorstellungen berichten (z. B. konkrete vs. abstrakte Ursachen). Die Verstehbarkeit der Hauterkrankung wurde in bisherigen qualitativen Studien noch nicht untersucht [30]. Daher werden im Anschluss Besonderheiten der Kategorie Kohärenz berichtet.

Der Fokus dieses Artikels liegt ferner auf der Analyse von Besonderheiten in Bezug auf Alters- und Berufsgruppen. Vor diesem Hintergrund werden diese im weiteren Verlauf im Rahmen von Gruppenvergleichen berichtet. Ergebnisse der Gruppenvergleiche folgender Kategorien werden dargestellt: Ursache, Kontrollierbarkeit, Konsequenzen, Emotionale Repräsentation.

### Ursache

Die Subkategorie *äußere Faktoren* der Ursachen zeigte sich mit einer Anzahl von 118 kodierten Textstellen am weitaus häufigsten. Die Einschätzung der Interviewten, dass äußere Einflüsse ursächlich für die Entstehung des eigenen Handekzems sind bzw. einen Einfluss auf den Erkrankungsverlauf haben, ließ sich zudem – mit Ausnahme von zwei Teilnehmenden – in den Transkripten aller Interviewten finden. Die zwei Interviewten, die keine äußeren Ursachen benannten (Gesundheits- und Krankenpflegerinnen), führten jeweils sowohl genetische als auch psychosoziale Faktoren als ursächlich für die Entstehung ihres Handekzems bzw. als Einflussfaktor auf. Ferner wurde innerhalb der einzelnen Interviews die Kategorie *äußere Faktoren* überwiegend mehrfach kodiert, da die Befragten häufig verschiedene äußere Einflussfaktoren nannten. Nur wenige Teilnehmende beschrieben lediglich einzelne vermutete äußere Ursachen.

Die Befragten benannten die wahrgenommenen äußeren Ursachen überwiegend konkret. So wurden bspw. relevante Arbeitsstoffe, wie Kühlschmiermittel (KSS), Desinfektionsmittel oder aber Feuchtigkeit mit der Entstehung oder Verschlimmerung des Handekzems in Verbindung gebracht. Vereinzelt wurde der Arbeitsplatz lediglich abstrakt als Ursache im Sinne eines auslösenden Faktors benannt, ohne Nennung konkreter Arbeitsstoffe bzw. Belastungen: „Meine Auslöser waren, weil ich arbeiten gegangen bin.“ (B24, Zerspanungsmechaniker, 35 Jahre) oder in der Form, dass die Ursache in der Tätigkeit bzw. dem Arbeitsplatz vermutet wird, es aber vage bleibt, welche Faktoren ursächlich für die Entstehung des Handekzems sind: „Wer weiß, was in den letzten Jahren auf diesen Zechen passiert ist. Das lässt sich ja nicht mehr nachvollziehen. Müsste man, wenn dann Bodenproben nehmen, aber ich weiß nicht, ja.“ (B1, 51 Jahre, Reifenmonteur/Vulkaniseur).

Nur wenige der 35 Interviewten (*n* = 6) nannten ausschließlich äußere Faktoren als Ursache für das eigene Handekzem. Alle anderen führten zumindest Ursachen aus einer oder aber mehreren weiteren Subkategorien auf. Als Auffälligkeit zeigte sich, dass auch diejenigen, die konkrete äußere Ursachen benannten (z. B. die Verwendung von Kühlschmiermitteln, Kontakt mit Desinfektionsmitteln), Ursachen weiterer Subkategorien (z. B. psychosoziale und genetische Ursachen) angaben. Die Entstehung des Handekzems wurde demzufolge in den allermeisten Fällen im Zusammenspiel verschiedener Ursachen bzw. Einflussfaktoren vermutet. Zudem zeigte sich, dass die äußeren Einflussfaktoren in den meisten Fällen mit dem Arbeitsplatz und den dort durchgeführten Tätigkeiten konnotiert waren. Vereinzelt wurden auch explizit außerhalb der Arbeit liegende äußere Faktoren benannt (z. B. Verschmutzungen im Rahmen der Ausübung eines Hobbys oder die Verwendung eines bestimmten Hautreinigungsmittels im häuslichen Umfeld). Zudem war eine Trennung zwischen den arbeitsplatzbezogenen Faktoren und außerhalb der Arbeit liegenden Einflüssen nicht immer möglich (z. B. vermehrtes Schwitzen im Sommer oder Heizungsluft im Winter).

Neben der Nennung konkreter äußerer Ursachen weisen die Antworten z. T. auch eine Einschätzung zur Bedeutung der Häufigkeit und Dauer von Hautbelastungen auf. So wurde das kumulative Auftreten einzelner Hautbelastungen oder auch die Kombination mehrerer Belastungen als Einflussfaktor bewertet:

„Also, ich glaube, dass die Häufigkeit der Belastungen schon eine Rolle spielt.“ (B13, 58 Jahre, Gesundheits- und Krankenpflegerin)

„Ja dieses Konglomerat an viel. Ne? Handschuhe tragen, Hände desinfizieren, auch dieses ständig –. Ich schwitze sofort unter den Handschuhen.“ (B20, 40 Jahre, Gesundheits- und Krankenpflegerin)

Ursachen wurden von nahezu allen Befragten als eigene Vorstellungen bzw. Vermutungen berichtet. Häufig wurden die Äußerungen mit einem Unsicherheitsfaktor belegt und mit Äußerungen wie „ich vermute“, „vielleicht“ oder „ich nehme stark an“ begonnen. Ein Befragter gab keine eigenen Vorstellungen an, sondern berichtete lediglich über die ärztlich vermutete Ursache. Die Äußerungen des Befragten legten nahe, dass er trotz eines arbeitskongruenten Verlaufs und der Assoziation, dass die berufliche Tätigkeit im Zusammengang mit dem Handekzem steht, keinen konkreten Einflussfaktor wahrgenommen hat. Die vermutete Ursache sei erstmalig im Rahmen der ärztlich durchgeführten Diagnostik durch den Arzt ausgesprochen worden: „Und dann meinte der Arzt das liegt an Kühlwasser, also an dem Bohrwasser“ (B6, 59 Jahre, CNC-Dreher). Die passive Haltung des Befragten spiegelte sich auch an anderer Stelle des Interviews wider. Hier wurde der Arbeitgeber als Initiator beschrieben, der dafür sorgte, dass der Befragte sich krankschreiben lässt. Die Einschätzung des Handekzems durch den Vorgesetzten schien eine andere zu sein, als die des Arbeitnehmers:

„Wieder eine Woche arbeiten und dann hat der Chef irgendwann gesagt: ‚Und wie sieht es aus?‘ Ja, ich sag ‚glaub geht es schon wieder‘ ‚Ja, das hat es so keinen Zweck. Musst du nochmal zum Arzt.‘ Und hat mich dann direkt zum Arzt auch geschickt. Und ja, jetzt bin ich mal hier gelandet.“ (B6, 59 Jahre, CNC-Dreher)

Auch bei anderen von den Interviewten geäußerten Ursachen war gelegentlich erkennbar, dass diese nicht vollständig als subjektive Vorstellungen der Befragten selbst zu werten sind. So wurden bspw. ärztliche Ursachenzuschreiben als plausibel erachtet und übernommen: „Da hat der Oberarzt dann halt schon recht, dass es irgendwas Genetisches, Veranlagung ist.“ (B5, 47 Jahre, Gesundheits- und Krankenpflegerin) Ferner gab eine Befragte an (B11, Gesundheits- und Krankenpflegerin, 54 Jahre), bei der das Handekzem plötzlich aufgetreten sei, dass sie keine Vermutungen habe, wodurch die Hauterkrankung sich entwickelt habe. Als Erklärung wurde eingeschoben, dass keine Änderungen der Produkte am Arbeitsplatz erfolgt seien. Man sei jedoch „ausgestattet mit tausenden von Cremes“, was „für die Haut auch nicht das Wahre“ sei. Die Interviewte hoffte, dass eine Ursache im Sinne einer Allergie gefunden werde, da sie dann etwas „Handfestes“ hätte, das sie meiden könne. Die Aussagen eines Befragten spiegelten wider, dass eine Unsicherheit bei der Einschätzung möglicher Ursachen bestand. So gab der Befragte einerseits an, dass er keine „Idee“ habe, wodurch die Hautveränderungen hervorgerufen wurden, er aber „ja Lackierer als Beruf“ sei. An anderer Stelle äußerte der Interviewte jedoch, dass er vermute, „dass es von den Einweghandschuhen kommt“ (B15, Lackierer, 35 Jahre).

Mehrere Befragte, die von konkreten äußeren Risikofaktoren/Ursachen berichteten, gaben in diesem Zusammengang auch an, welche unmittelbaren Folgen der Kontakt mit den als Hautrisiko erachteten Substanzen hatte. So sei eine Veränderung der Haut nach der Verwendung entsprechender Arbeitsstoffe (z. B. Bremsenreiniger und Waschbenzin) festgestellt worden: „Die Haut wurde sofort spröde und rissig.“ (B4, Werkzeugmacher, 59 Jahre). Im weiteren Verlauf des Interviews äußerte dieser Befragte jedoch, dass die Hautprobleme erst später entstanden seien und ergänzte, dass dies durch die geforderte Händedesinfektion in Rahmen der SARS-CoV-2-Pandemie hervorgerufen worden sei. Ein Befragter gab zudem an, dass er in der Vergangenheit, insbesondere im Winter, bereits „Probleme“ gehabt habe. Die Haut sei „dünner“ geworden. Es sei allerdings zu diesem Zeitpunkt noch kein „Ekzem“ gewesen (B16, Metallbauer, 45 Jahre).

Als weiterer Aspekt fiel zudem auf, dass Befragte z. T. äußere Einflussfaktoren nicht nur konkret benannten, sondern teilweise auch in einen zeitlichen Rahmen einordneten. Die zeitliche Dimension zeigte sich in der Form, dass Interviewte bspw. der Auffassung waren, dass die eigentliche Ursache der Erkrankung eher in früheren Hautbelastungen vermutet wurde. Frühere Hautbelastungen wurden z. B. als initialer Faktor für die Entstehung des Handekzems gedeutet:

„Ich würde fast behaupten, dass diese extremen Reinigungsmittel in meiner Fleischerlehre diese Sachen halt oder Probleme, die ich jetzt immer noch habe, vielleicht begünstigt haben, weil es gab da starke Laugen, um Rauchschränke zu reinigen, Eiweißfettlöser, aber keine Handschuhe.“ (B1, 51 Jahre, Reifenmonteur/Vulkaniseur)

„Wohl muss ich dazu sagen, wir haben schon in der Werkstatt Arbeitsstoffe, ich sage mal, wie früher hatten wir ein Waschbenzin. Oder diese Aceton oder diese so auch Bremsenreiniger, wenn man den auf die Hände bekam, dann, das war als, wie soll ich sagen, die Haut wurde sofort spröde und rissig.“ (B4, 59 Jahre, Werkzeugmacher)

Auf der anderen Seite scheint es aus Sicht mehrerer Befragter so zu sein, dass Veränderungen des Arbeitsplatzes innerhalb der vergangenen Jahre und daraus teilweise resultierende, neue Anforderungen zu einer vermehrten Hautbelastung geführt haben. Dies habe dazu beigetragen, dass das Handekzem entstanden sei bzw. zu einer Verschlimmerung geführt:

„Ich glaube, ja, ich sage mal: Die Anzahl des Handschuhtragens ist, glaube ich, mehr geworden als früher. Ja, man zieht einfach mehr an. Das lässt sich nicht vermeiden. Manchmal auch doppelt. Also, das ist eine größere Belastung für die Haut, ohne Frage, weil die Abstände zwischen dem Handschuhtragen auch immer kürzer werden.“ (B13, 58 Jahre, Gesundheits- und Krankenpflegerin)

„Und denn, ja da haben wir ein neues Mittel gekriegt. Auch Reinigungsmittel für die Motoren. Früher hatten wir ja so einen Waschboy, da haben wir die Teile alle mit gewaschen und heute kommt alles aus der Sprühdose. Mit, damit werden die Teile gewaschen. Aber die sind so, ja, wie soll ich sagen, fast ätzend.“ (B34, 63 Jahre, Servicetechniker in Kfz-Werkstatt)

Hinsichtlich der Häufigkeit wurden mit deutlichem Abstand *psychosoziale Faktoren* nach *äußeren Faktoren *am zweithäufigsten kodiert (29 Kodierungen). Von den Befragten wurde häufig „Stress“ als ursächlicher oder beeinflussender Faktor genannt. Der Begriff Stress wurde dabei von einigen Interviewten als allgemein Faktor verbalisiert, von anderen anhand konkreter Beispiele (z. B. Zeitdruck).

### Kohärenz

In Bezug auf die Verstehbarkeit des Handekzems wurde deutlich, dass sowohl bei Interviewten, die ausschließlich äußere Ursachen (und damit tendenziell beeinflussbare Faktoren) für die Entstehung des Handekzems annahmen, Äußerungen gefunden wurden, die Aspekte von Schicksalshaftigkeit und Gerechtigkeit enthielten, als auch bei denjenigen, die genetische Faktoren benannten:

„Das kann ich halt gar nicht sagen, weil bei mir ist es so, ich stehe frühmorgens auf, okay, habe ich Glück oder habe ich Pech?“ (B2, Gesundheits- und Krankenpflegerin, 23 Jahre – keine äußeren Faktoren)

„Ja Herrgott, mich hat es halt getroffen. Manche trifft es nicht. Mich hat es jetzt erwischt.“ (B3, Gesundheits- und Krankenpflegerin, 60 Jahre – keine genetischen Faktoren)

„Weil man dann sieht, die anderen arbeiten den ganzen Tag ohne Handschuhe und ich selber muss mir Gedanken machen, wann creme ich meine Hände ein, welche Handschuhe ziehe ich zu der Arbeit an, wie schütze ich meine Hände am besten?“ (B32, Kfz-Mechatroniker, 22 Jahre, äußere und genetische Faktoren)

Aussagen einiger Befragter ließen zudem eine Ambivalenz zwischen einer Verstehbarkeit und einer Nicht-Verstehbarkeit der eigenen Erkrankung erkennen. So äußerte eine Befragte, die eine erbliche Veranlagung und Stress als Ursachen aufführte: „Klar, es ist Neurodermitis bedingt, aber, ich sag mal so, auch irgendwie nicht.“ (B2, Gesundheits- und Krankenpflegerin, 23 Jahre). Die Befragte begründete dies im weiteren Verlauf insofern, dass ihr die Neurodermitis bekannt sei und dadurch auch z. T. verstehbar, dass sie ein Handekzem habe. Da sich Tätigkeiten seit der Ausbildung nicht geändert hätten, sei jedoch nicht nachvollziehbar, warum das Ekzem an den Händen entstanden sei. Demgegenüber sei eine Allergie, die jedoch nicht bestehe, aus Sicht der Interviewten eine nachvollziehbare Erklärung.

Auch bei Interviewten, die konkrete Ursachen für ihr Handekzem benannt haben, bestand in Einzelfällen eine Ambivalenz in Bezug auf die Verstehbarkeit/Nachvollziehbarkeit des Handekzems. Hierbei schien es z. T. so, dass nicht nachvollziehbar sei, warum bei gleichbleibender Belastung/keiner Veränderung der bestehenden Belastungen, die Hauterkrankung unvermittelt aufgetreten ist:

„‚Und da ist, Sie reagieren auf irgendwas.‘ Sage ich: ‚Ich kann doch nicht auf irgendwas reagieren, ich habe vierzig Jahre auf nichts reagiert. Plötzlich reagiere ich.‘“ (B4, Werkzeugmacher, 59 Jahre)

### Betrachtung von Alters- und Berufsgruppen

Im Folgenden werden Auffälligkeiten für die Hauptkategorie Ursachen und die Subkategorien Allergie, psychosoziale Faktoren und Lebensstil sowie die Kategorien Kontrollierbarkeit, Konsequenzen und emotionale Repräsentationen berichtet, die sich im Vergleich der Alters- bzw. Berufsgruppen zeigten.

#### Ursache – Allergie

Bei Befragten aus allen drei Berufsgruppen waren Äußerungen zu finden, in denen eine allergische Reaktion als mögliche Ursache bzw. Teilursache für die Entstehung des Handekzems vermutet wurde. Hinsichtlich der Häufigkeit zeigte sich, dass bei Gesundheitsberufen und körpernahen Dienstleistungsberufen (*n* = 13) Aussagen von drei Interviewten in diese Kategorie kodiert wurden. Bei den Metallberufen und weiteren Handwerksberufen (*n* = 19) wurden Aussagen von fünf Befragten dieser Kategorie zugeordnet. In der Gruppe der Verkäufer*innen (*n* = 3) wurden Aussagen von zwei Befragten kodiert.

Es zeigte sich z. T. sowohl in Aussagen von befragten Pflegekräften als auch in der Gruppe der Befragten aus den Metallberufen und der Verkäuferinnen, dass eine Allergie zwar als (Teil)ursache in der Vergangenheit vermutet wurde, im Rahmen durchgeführter Allergietests jedoch keine Allergie festgestellt worden sei. Die Glaubhaftigkeit der Ergebnisse jeweiliger Allergietests scheint jedoch unterschiedlich ausgeprägt zu sein. Auf der einen Seite führte ein negativer Allergietest bei mehreren Befragten offenbar dazu, dass eine Allergie als Ursache ausgeschlossen oder zumindest als nicht mehr wahrscheinlich angesehen wurde:

„Habe ich erst vermutet, aber ich reagiere ja nicht auf das Desinfektionsmittel.“ (B20, 40 Jahre, Gesundheits- und Krankenpflegerin)

„Aber die habe ich ja dann auch, exakt dieses Paar von dem Tag, am Anfang des Jahres in (Name der Stadt) als Allergietest auf den Rücken geklebt gehabt und das war nichts.“ (B1, 51 Jahre, Reifenmonteur)

Auf der anderen Seite ist die Konsequenz eines negativen Allergietests nicht unweigerlich, dass Befragte eine Allergie ausschließen. So könnte ein negativer Allergietest auch das Resultat daraus sein, dass die allergieauslösenden Stoffe nicht getestet wurden und somit eine Allergie weiterhin als Ursache vermutet werde:

„Das ist ja so eine Sache, viele Sachen werden ja gar nicht getestet.“ (B16, 45 Jahre, Metallbauer)

Eine vergleichbare Einschätzung ließ sich auch bei einer befragten Verkäuferin finden. Hier scheint es bislang keine Hinweise für eine vermutete Allergie gegenüber Metallen oder Stäuben zu geben. Die Vermutung, dass die Hautveränderungen daraus resultieren, dass die Haut auf einen Arbeitsstoff „allergisch“ reagiert, besteht weiterhin:

„Gut, dann ist es wahrscheinlich auf dem Metall. Entweder bist du gegen das Metall allergisch oder auch auf den-, der Schmutz, der da drauf ist.“ (B36, 60 Jahre, Verkäuferin)

#### Ursache – psychosoziale Faktoren

Von den Interviewten wurden psychosoziale Einflussfaktoren überwiegend unter dem Begriff „Stress“ verbalisiert. Es stach hervor, dass die Teilnehmenden aus den Gesundheitsberufen und körpernahen Dienstleistungsberufen beruflichen Stress überwiegend konkret mit der Entstehung bzw. der Verschlechterung des Handekzems in Verbindung brachten:

„Also, bei mir persönlich jetzt, ist das schon so, dass ich viel, sage ich mal, ich würde sagen, schon teilweise auch psychisch bedingt ist. Also, durch Stress einfach, dass ich, dass das auf meiner Haut auch ausbricht.“ (B2, Gesundheits- und Krankenpflegerin, 23 Jahre)

„(…) und Stress, ich gehe sogar fest dahin, dass das mehr so der Stress war.“ (B10, Friseur, 45 Jahre)

Die Befragten, die in Metallberufen und weiteren handwerklichen Berufen tätig waren, verwendeten ebenso den Begriff ‚Stress‘, um die vermuteten psychosozialen Ursachen zu benennen. Im Vergleich zu anderen Berufsgruppen waren die Aussagen hier jedoch überwiegend vage und eher im Sinne einer Vermutung:

„Ich weiß nicht, ich meine, stressig waren meine letzten Lebensjahre sowieso, sehr. Ich weiß nicht, ob der Stress vielleicht mit reingespielt hat.“ (B14, Modellbauerin, 51 Jahre)

„Also keine Ahnung, ob da vielleicht irgendwie Stress noch zustande kam.“ (B33, Industriemechanikerin, 33 Jahre)

#### Ursache – Lebensstil

In wenigen Interviews wurden Äußerungen gefunden, die darauf hindeuteten, dass der Lebensstil als mögliche (Teil)ursache für die Entstehung des Handekzems oder den Verlauf angenommen wurde. Im Vergleich der Berufsgruppen zeigte sich die Besonderheit, dass entsprechende Äußerungen lediglich in den Interviews von drei Befragten aus den Metallberufen und anderen handwerklichen Berufen gefunden wurden. Dabei wurde in allen drei Fällen Ernährung als Einflussfaktor genannt. Es habe aus Sicht der Befragten entweder das Essen von „etwas Falschem“ oder die Ernährung allgemein zum Handekzem beigetragen. Ein weiterer Befragter vermutete, dass das Weglassen bestimmter Lebensmittel zu einer Verbesserung des Handekzem geführt haben könnte.

#### Kontrollierbarkeit

Tendenziell wurden bei der jüngsten Altersgruppe (18–35 Jahre) weniger Äußerungen zur Kategorie *erfolgreiche Kontrolle* kodiert, aber im Vergleich zu den beiden anderen Altersgruppen (36–50, 51–65 Jahre) auch weniger Äußerungen zur *fehlenden Kontrolle*. Inhaltlich zeigte sich, dass in allen Altersgruppen verschiedene Aspekte zu einer erlebten Kontrolle des Handekzems geführt haben. Hierbei wurden sowohl eigenes Verhalten (Ablenkung durch andere Dinge), als auch veränderte Ernährungsgewohnheiten (Weglassen von Südfrüchten) sowie die ärztliche Versorgung und Gabe von Medikamenten genannt (z. B. Verwendung von Cortison).

Im Vergleich der Berufsgruppen ließ sich erkennen, dass Aussagen, die auf eine Selbstwirksamkeitsüberzeugung in Bezug auf den Einfluss auf die Entwicklung des Handekzems hindeuten, lediglich bei einem der Befragten aus den Metall- und Handwerksberufen tendenziell und bei keiner der Verkäufer*innen gefunden wurden. Bei den Gesundheitsberufen und körpernahen Dienstleistungsberufen wurden Aussagen von 6 Interviewten in dieser Kategorie kodiert:

„Und je länger das war, umso besser konnte man einschätzen, jetzt fängt es an, jetzt wird es intensiv. Jetzt kann ich es noch tolerieren. Ich kann noch selber was machen. Oder ich muss aktiv beim Arzt werden.“ (B3, Gesundheits- und Krankenpflegerin, 60 Jahre)

Auf der anderen Seite wurden bei den Teilnehmenden aus den Metall- und Handwerksberufen keine Aussagen gefunden, die auf eine fehlende Selbstwirksamkeit hindeuten. Bei einer Gesundheits- und Krankenpflegerin sowie bei einem Befragten aus einem körpernahen Dienstleistungsberuf wurden Aussagen zu fehlender Selbstwirksamkeit kodiert. Eine gewisse Ambivalenz (sowohl Selbstwirksamkeit als auch fehlende Selbstwirksamkeit) ist bei der genannten Gesundheit- und Krankenpflegerin zu erkennen. Beim Vergleich der dazugehörigen Aussagen lässt sich jedoch erkennen, dass die Selbstwirksamkeit dort vorhanden ist, wo durch eigenes Verhalten eine Einflussmöglichkeit gesehen wird – in diesem Fall bei der Vermeidung des Kratzens trotz bestehendem Juckreiz. Fehlende Selbstwirksamkeit wird dann erlebt, wenn die Einflussmöglichkeiten durch vorgegebene und erforderliche Arbeitsabläufe eingeschränkt sind:

„Gerade die Pflege der Hände geht so ein bisschen bei unserer Arbeit unter. Weil, wenn es schnell gehen muss, kann ich nicht erst sagen ‚Oh Moment, ich muss jetzt noch 15 min warten.‘“ (B5, Gesundheits- und Krankenpflegerin, 47 Jahre)

#### Psychische Konsequenzen

Über alle Altersgruppen hinweg waren psychische Konsequenzen der Erkrankung im sozialen Kontext der Interviewten zu erkennen. So zeigten sich im familiären Umfeld, im Freundeskreis, aber auch im beruflichen Kontext und in Alltagssituationen, wie dem Bezahlen im Supermarkt, Situationen, die zu psychischen Konsequenzen bei den Betroffenen geführt haben. Bei zwei Interviewten aus der jüngsten Altersgruppe (18–35 Jahre) ließen sich Aussagen finden, in denen die Berührung des Partners bzw. des eigenen Kindes mit der ekzematösen Haut der Hände als unangenehm beschrieben wurde:

„(…) dann möchte ich auch nicht meinen Partner irgendwie anfassen oder so, weil mir das halt dann auch sehr unangenehm ist.“ (B25, Altenpflegerin, 26 Jahre)

#### Finanzielle Konsequenzen

Von Befragten aus den Gesundheitsberufen wurden keine finanziellen Konsequenzen genannt. Bei den Interviewten, die in Metallberufen und weiteren handwerklichen Berufen tätig waren, führten drei Personen mögliche finanzielle Folgen ihres Handekzems auf. Die drei männlichen Interviewten deckten alle drei Altersgruppen ab. In den Aussagen schienen potenzielle Mehrkosten durch dauerhafte Behandlung und die finanzielle Absicherung der Familie wichtige Aspekte zu sein:

„Wie sieht denn das nachher aus, wenn ich in Rente gehe? Ist die BG dann auch noch für mich da, und zahlt mir eventuell meine Pflegemittel? Oder muss ich dann in meine eigne Hosentasche greifen?“ (B9, Montagearbeiter, 62 Jahre)

„Ich bin der, der das Geld, sage ich mal, nach Hause bringt.“ (B24, Zerspanungsmechaniker, 35 Jahre)

#### Berufliche Konsequenzen

In den beiden Berufsgruppen „Verkäufer*innen“ sowie „Gesundheitsberufe und körpernahe Dienstleistungsberufe“ sind Aussagen der Befragten zu finden, die zeigen, dass die aus Emotionen wie „Scham“ hervorgehende psychische Konsequenz „Verstecken der Hände“ oder die Sorge darüber, was Mitmenschen am Arbeitsplatz (z. B. Kund*innen und Patient*innen) denken, auch eine berufliche Konsequenz des Handekzems sein kann:

„(…) wenn ich mit Kunden dann gesprochen habe, beraten habe. Ich habe dann halt es anders anders handhabt so, wenn die ganz schlimm waren, dass ich dann auch gar nicht mehr viel Sachen gezeigt habe, sondern aus der weiten Entfernung quasi nur darauf gezeigt habe (…).“ (B35, Verkäuferin, 36 Jahre)

„Da habe ich halt auch manchmal auf Arbeit immer so ein bisschen Bedenken gehabt, wenn die Eltern das sehen, gerade wenn man mit Frühchen arbeitet und kleinen Kindern und Säuglingen.“ (B3, Gesundheits- und Krankenpflegerin, 60 Jahre)

Bei einem Teilnehmenden aus einem Metallberuf führten hingegen nicht die eigenen Gedanken oder Schamgefühl zu einem Verstecken der Hände, sondern Kommentare der Arbeitskollegen:

„Dann fingen die anderen auch an, die Arbeitskollegen. Und die sitzen in einem Frühstücksraum und denn war auch nicht schön. Da sagten sie: ‚Setz dich woanders hin. Das sieht nicht schön aus.‘ Ich sage: ‚Ich kann aber auch nichts daran machen. Es ist eben so.‘ Dann haben die gesagt: ‚Dann zieh irgendwas über.‘“ (B34, Servicetechniker in Kfz-Werkstatt, 63 Jahre)

Ferner waren berufliche Konsequenzen in den verschiedenen Berufsgruppen zu finden, die ausgehend vom Tätigkeitsfeld divergierten. So war insbesondere bei Befragten aus den Pflegeberufen eine unmittelbare Konsequenz die Sorge vor Hygieneproblemen, infolge der mangelnden Desinfizierbarkeit der Hände sowie die Vermeidung des direkten Hautkontaktes mit Patient*innen:

„Das ist so ein Gewissenskonflikt Schmerz oder Hygiene. Was ja, schwierig ist.“ (B3, Gesundheits- und Krankenpflegerin, 60 Jahre)

Eine mehrfach auftretende berufliche Konsequenz des Handekzems bei Interviewten aus den Metall- und Handwerksberufen war eine Einschränkung bei der Ausführung der Tätigkeit, z. B. durch Schmerzen oder fehlende Motorik:

„Oh, Scheiße, es tut jetzt so weh, dass ich nichts mehr anfassen kann.“ (B6, 59 Jahre, CNC-Dreher)

„Manchmal sind bei mir Materialstücke, beziehungs- Werkzeuge einfach so von Hand gefallen, weil ich die einfach nicht normal greifen konnten.“ (B24, Zerspanungsmechaniker, 35 Jahre)

Die Sorge, durch Arbeitskolleg*innen und/oder Vorgesetzte als unzuverlässig wahrgenommen zu werden, zeigte sich in Aussagen von Befragten aus den Gesundheitsberufen und köpernahen Dienstleistungsberufen ebenso wie bei Interviewten aus den Metall- und Handwerksberufen. Über alle Berufsgruppen hinweg berichteten die Befragten über Arbeitsunfähigkeitszeiten und zudem über Sorgen und Unsicherheiten hinsichtlich der beruflichen Zukunft:

„Die hat das schon als grenzwertig eingestuft und hat mich dann auch gefragt, ob ich vielleicht schon mal dran gedacht hätte, meinen Beruf zu wechseln. Habe ich aber nicht.“ (B13, Gesundheits- und Krankenpflegerin, 58 Jahre).

„Und so, ich kann mir das gar nicht vorstellen, im Büro zu sitzen oder ja, das ist eigentlich nicht mein Ding. Aber wenn das jetzt hier besser ist und ich dann wirklich keine Probleme habe, dann weiß ich halt auch nicht, wenn das in zwanzig Jahren nochmal kommt, dann ist das nochmal was Anderes, in einen neuen Beruf einzusteigen oder so.“ (B32, Kfz-Mechatroniker, 22 Jahre)

#### Emotionale Repräsentation

Hinsichtlich der Emotionen berichteten wenige Befragte, dies jedoch über alle Altersgruppen hinweg, über Traurigkeit im Zusammenhang mit dem Handekzem. In allen drei Altersgruppen wurde Angst als Begrifflichkeit im Zusammenhang mit dem Handekzem genannt. In der Altersgruppe der 18–35 Jahre alten Befragten tauchte dieser Begriff jedoch lediglich einmal auf und wurde vor dem Hintergrund genannt, dass Angst davor bestehe, dass aufgrund von Arbeitsunfähigkeit die Zuverlässigkeit durch den Arbeitgeber in Frage gestellt werde. Bei der ältesten Altersgruppe wurde Angst von mehreren Befragten – teils mehrfach – genannt. Hier war eine Tendenz in Richtung einer Zukunftsangst und einer Angst vor einem Arbeitsplatzverlust erkennbar.

„Weil ich bin in nem gewissen Alter, wo man natürlich auch ein bisschen Angst hat, dass man Arbeitsplatz verliert.“ (B6, CNC-Dreher, 59 Jahre)

## Diskussion

Ziel dieser qualitativen Studie war die Untersuchung subjektiver Krankheitstheorien von Patient*innen mit berufsbedingten Handekzemen mit dem Fokus auf Besonderheiten in verschiedenen Alters- und Berufsgruppen. Da berufsbedingte Handekzeme grundsätzlich in allen Altersgruppen auftreten können sowie vor dem Hintergrund möglicher unterschiedlicher allgemeiner Erfahrungen mit Erkrankungen und gesundheitlichen Einschränkungen in verschiedenen Altersphasen, wurden Interviews mit Patient*innen aus der Altersspanne 18–65 Jahren avisiert. Die Aufteilung im Rahmen der Analyse erfolgte in drei Altersgruppen (18–35, 36–50, 51–65 Jahre). Interviews konnten mit Frauen und Männern aus allen drei Altersgruppen geführt werden. Der Fokus der befragten Berufsgruppen lag auf den besonders häufig betroffenen Gesundheits- und Metallberufen. Auffälligkeiten hinsichtlich möglicher Geschlechterunterschiede werden dargestellt, jedoch aufgrund der Kongruenz mit den genannten Berufsfeldern nicht gesondert analysiert.

Die befragten Patient*innen äußerten überwiegend komplexe Vorstellungen über die Ursachen der Handekzeme bzw. hinsichtlich möglicher Risikofaktoren. So wurden von den meisten Befragten Ursachen verschiedener Subkategorien (z. B. äußere arbeitsplatzbezogene Risiken bei gleichzeitig bestehender genetischer Hautempfindlichkeit) genannt. Die Komplexität der subjektiven Vorstellungen spiegelt die klinisch häufig auftretenden Mischdiagnosen (z. B. irritativ verursachtes Handekzem bei atopischer Disposition) wider [35]. Die oftmals konkret benannten äußeren Einflüsse verdeutlichen, dass überwiegend klar benennbare Arbeitsstoffe mit der Entstehung des Handekzems in Verbindung gebracht wurden. Die jedoch teils lediglich vage Vermutung, dass die Erkrankung durch die Arbeit entstanden sei, lässt sich möglicherweise dadurch erklären, dass von den Betroffenen ein arbeitskongruenter Verlauf des Ekzems festgestellt wurde, der für einen Zusammengang mit der Arbeitstätigkeit spricht, jedoch (noch) keine konkreten Arbeitsstoffe identifiziert werden konnten. Ferner besteht vor dem Hintergrund des Settings, in dem die Interviews durchgeführt wurden, die Möglichkeit, dass der Fokus stärker auf die selektive Suche nach Hinweisen für eine berufliche Kausalität gelegt wird. In diese Einschätzung reihen sich die Aussagen einer befragten Gesundheits- und Krankenpflegerin (B5, 47 Jahre) ein, die einerseits vermutete, dass das Handekzem nichts mit der Arbeit zu tun habe, an anderer Stelle jedoch die Verwendung von Desinfektionsmitteln als mögliche Ursache sah. Ein Kausalitätsbedürfnis zeigte sich nicht nur hinsichtlich des Berufs, sondern z. T. auch hinsichtlich der subjektiven Erklärung, dass eine Allergie ursächlich für das Handekzem sei. Die aus Sicht mehrerer Interviewter plausible Erklärung war so verfestigt, dass auch ein negativer Allergietest die Vorstellung nicht veränderte. Ähnliches berichteten auch Mollerup et al. [27]. Als Auffälligkeit wurde festgestellt, dass von zwei der befragten Verkäuferinnen (*n* = 3) eine Allergie als Ursache vermutet wurde. Wenngleich dies aufgrund der geringen Zahl der interviewten Verkäuferinnen zurückhaltend interpretiert werden muss, kann es darauf hindeuten, dass insbesondere in den Berufen mit geringen irritativen Reizen bei trotzdem wahrgenommenen Berufsbezug eine Allergie eine plausibles Erklärungsmuster darstellt und ein Kausalitätsbedürfnis befriedigen kann. In diese Argumentation reiht sich ein, dass bei einer der Befragten im Rahmen eines durchgeführten Allergietests keine Allergien festgestellt worden seien. Kontaktstoffe wurden trotz dessen weiterhin von der Interviewten als Allergenquelle vermutet.

Einige Interviewte unterschieden zwischen manifesten Ursachenzuschreibungen für das Handekzem und den Risikofaktoren, die zwar zu einer Hautschädigung (z. B. dünner oder spröder gewordene Haut) geführt haben, ohne dass jedoch von einer Erkrankung gesprochen wurde. Dies deutet darauf hin, dass wahrgenommene Symptome unterschiedlich bewertet werden und die Grenze zur Erkrankung interindividuell unterschiedlich erlebt wird. Es verdeutlicht, dass eine Erkrankung („disease“, in diesem Fall ein Handekzem) aus ärztlicher Perspektive bereits vorliegen kann, obwohl in der subjektiven Wahrnehmung der betroffenen Person die Hautveränderungen nicht als Krankheit wahrgenommen werden („illness“; [17]) und vice versa. Die Bedeutung dieser Diskrepanz für die Interaktion zwischen Patient*in und Therapeut*in/Ärzt*in liegt auf der Hand: Wird bspw. ein behandlungsbedürftiges Handekzem ärztlich diagnostiziert, von Seiten des/der Patient*in den Hautveränderungen jedoch (noch) kein Erkrankungswert beigemessen, kann sich dies unmittelbar auf die Adhärenz auswirken sowie darüber hinaus bereits auf die Wahrscheinlichkeit, überhaupt einen Arzt/eine Ärztin aufzusuchen.

Ambivalenzen waren auch in anderen Bereichen zu erkennen. So nahmen mehrere Interviewte das Handekzem als teilweise verstehbar bzw. nachvollziehbar wahr. Gleichwohl waren Elemente von einer Nicht-Verstehbarkeit in den Aussagen vorzufinden. Dies erklärt sich vor dem Hintergrund, dass z. B. eine erblich bedingte Neigung zur Neurodermitis zwar grundsätzlich die Entstehung einer entzündlichen Hauterkrankung erklärt. Die Nicht-Verstehbarkeit bezieht sich auf das konkrete Handekzem, das bei gleichbleibender Hautbelastung plötzlich aufgetreten ist.

Die Betrachtung von Alters- und Berufsgruppen zeigte, dass teilweise Unterschiede auf der qualitativen Ebene bestehen. So stellten bspw. Pflegekräfte eher einen konkreten Zusammenhang mit beruflichem Stress und der Hauterkrankung her. Bei mehreren Interviewten aus den Metallberufen spielte Stress ebenso eine Rolle. Beim konkreten Zusammenhang mit der Hauterkrankung waren die Äußerungen jedoch eher vermutend. Aussagen, die eine Selbstwirksamkeit in Bezug auf die Kontrollierbarkeit des Handekzems nahelegen, wurden eher bei den interviewten Pflegekräften gefunden, als bei den Befragten der Metallberufe. Eventuelle Zusammenhänge könnten im Rahmen nachfolgender, quantitativer Untersuchungen näher betrachtet werden. Das ausschließliche Vorfinden finanzieller Konsequenzen bei drei männlichen Interviewten aus den Metall- und Handwerksberufen, könnte ein möglicher Hinweis auf die Bedeutung der sozialen Rolle des „männlichen Ernährers“ sein und dass die Sorge vor dem Verlust des Berufs als bedrohlicher wahrgenommen wird. Bathe et al. [1] fanden in ihrer Studie zudem heraus, dass Frauen die Möglichkeit einer Berufsaufgabe eher in Betracht zogen als Männer. Inwiefern weitere Faktoren in die Sorge vor finanziellen Konsequenzen einfließen (z. B. empfundene Arbeitsplatzsicherheit in unterschiedlichen Berufsgruppen) lässt sich nicht beurteilen. Die in einigen Kategorien vorgefundenen Unterschiede zwischen den untersuchten Gruppen, in Bezug auf das Alter und die Berufe, lassen allerdings darauf schließen, dass die soziodemografischen Merkmale Alter, Geschlecht und Beruf eine Bedeutung bei der Ausprägung subjektiver Krankheitstheorien bei beruflich Hauterkrankten haben können. Ob und inwiefern weitere Merkmale wie z. B. der Bildungsgrad, Einkommensverhältnisse und Migrationshintergrund bei der Ausbildung subjektiver Krankheitstheorien beruflich Hauterkrankter – insbesondere auf qualitativer Ebene – bedeutsam sind, sollte in weiteren Studien untersucht werden.

Hinsichtlich der einzelnen Kategorien ließen sich z. T. Überschneidungen feststellen. So konnten u. a. private Konsequenzen gleichwohl auch andere Konsequenzen beinhalten (z. B. physische und psychische). Ferner waren berufliche Konsequenzen teilweise z. B. mit physischen Konsequenzen überlappend. Neben Überschneidungen innerhalb einer Hauptkategorie konnte auch zwischen Hauptkategorien eine unvollständige Trennschärfe festgestellt werden. Berufliche Konsequenzen bspw. können mit emotionalen Repräsentationen (z. B. Angst) einhergehen.

## Stärken und Limitationen

Als Limitation der Studie ist die Beschränkung auf Patient*innen der stationären (tertiären) Individualprävention zu nennen. Inwiefern die hier dargestellten Ergebnisse auf Patient*innen mit milderen Formen von Handekzemen bzw. Hautveränderungen übertragbar sind, bleibt offen. Ferner lassen sich keine Aussagen über andere betroffene Berufsgruppen treffen, die im Rahmen dieser Studie nicht interviewt wurden. Gleichzeitig ermöglichten die Einschränkungen auf stationäre Patient*innen und einzelne Berufsgruppen eine bessere Vergleichbarkeit innerhalb der Studie. Eine weitere Limitation bildet das Setting, in dem die Studie durchgeführt wurde. Beide Interviewer*innen sind in die Beratung und Schulung von Patient*innen in der stationären Maßnahme involviert, so dass möglicherweise eine soziale Erwünschtheit bei Aussagen der Befragten eine Rolle spielen könnte. Eine Stärke der hier berichteten Studie ist die erstmalige Verwendung des weit verbreiteten theoretischen CSM-Rahmenmodells im Rahmen berufsdermatologischer qualitativer Forschung.

## Fazit für die Praxis

- Alle Berufsgruppen (z. B. Ärzt*innen, Psycholog*innen, weitere Therapeut*innen Gesundheitspädagog*innen sowie auch Mitarbeiter*innen der Unfallversicherungsträger), die in Maßnahmen der Individualprävention berufsbedingter Handekzeme involviert sind, sollten in der Interaktion mit Patient*innen subjektive Krankheitsvorstellungen der Betroffenen berücksichtigen.
- Wenn Schwierigkeiten in der Kommunikation mit Patient*innen wahrgenommen werden, sollte eruiert werden, ob unterschiedliche Vorstellungen über das Handekzem (z. B. hinsichtlich der Ursachen, der Kontrollierbarkeit oder möglicher Konsequenzen) bestehen. Wenn möglich, sollten die Vorstellungen konkret erfragt werden.
- Mögliche Unterschiede auf Grundlage soziodemografischer Merkmale (z. B. Alter, Berufsgruppen, Geschlecht) sollten bedacht werden.
- Gleichwohl sollten diese möglichen Besonderheiten bzw. Unterschiede vor dem individuellen Hintergrund betrachtet werden und nicht zu pauschalen Aufteilungen einzelner Gruppen führen.
